# Anthracycline rechallenge using pegylated liposomal doxorubicin in patients with metastatic breast cancer: a pooled analysis using individual data from four prospective trials

**DOI:** 10.1038/sj.bjc.6605961

**Published:** 2010-10-26

**Authors:** S-E Al-Batran, M Güntner, C Pauligk, M Scholz, R Chen, B Beiss, S Stopatschinskaja, W Lerbs, N Harbeck, E Jäger

**Affiliations:** 1 Klinik für Onkologie und Hämatologie am Krankenhaus Nordwest, Krankenhaus Nordwest, Steinbacher Hohl 2-26, 60488 Frankfurt am Main, Germany; 2 Trium Analysis Online GmbH, München, Germany; 3 Merck, Kenilworth, NJ, USA; 4 Breast Center, University of Cologne, Cologne, Germany

**Keywords:** pegylated liposomal doxorubicin, metastatic breast cancer, anthracycline, rechallenge

## Abstract

**Background:**

The aim of this study was to determine the activity of anthracycline rechallenge using pegylated liposomal doxorubicin (PLD) in patients with metastatic breast cancer (MBC) previously treated with conventional anthracyclines.

**Methods:**

Pooled individual data from four prospective trials were used, and the primary end point of the pooled analysis was clinical benefit rate (CBR). The studies comprised 935 patients, of whom 274 had received PLD in the metastatic setting after prior exposure to conventional anthracyclines (rechallenge population).

**Results:**

The majority of patients were heavily pretreated. Previous anthracycline therapy was administered in the adjuvant (14%) or metastatic setting (46%), or both (40%). The overall CBR from rechallenge with PLD was 37.2% (95% CI, 32.4–42.0). In univariate analyses, the CBR was significantly higher in patients with less exposure to prior chemotherapy, in taxane-naive patients, and in patients with a favourable Eastern Cooperative Group performance status of 0 *vs* 1 *vs* 2 (53.3 *vs* 35.5 *vs* 18.2% *P*<0.001). In multivariate analyses, performance status proved to be the only independent predictor of the CBR achieved with PLD rechallenge (*P*=0.038). There was no statistically significant difference in CBR regarding the setting, cumulative dose of and/or resistance to prior anthracyclines, or time since prior anthracycline administration.

**Conclusion:**

Anthracycline rechallenge using PLD is effective in patients with MBC who have a favourable performance status, regardless of setting, resistance, cumulative dose or time since prior conventional anthracycline therapy.

In Europe and the United States, breast cancer remains the number one diagnosed cancer in women (incidence rate approximately 28%); and in Europe, it is the leading cause of death from cancer in women (Ferlay *et al*, 2007; Jemal *et al*, 2009). Though the majority of women with early-stage disease receive adjuvant systemic treatment to prevent disease recurrence, approximately 30–70% of patients develop metastatic breast cancer (MBC) (Early Breast Cancer Trialists' Group, 2005; Cardoso *et al*, 2009). MBC patients represent a very heterogeneous population, and the number of available therapies for MBC is rapidly growing. Anthracyclines and taxanes remain the most active cytotoxic agents in the treatment of this disease, and as such, have been widely integrated into adjuvant regimens for early-stage breast cancer (Early Breast Cancer Trialists' Group, 2005; Beslija *et al*, 2009; Cardoso and Castiglione, 2009; Cardoso *et al*, 2009).

Clinicians still face a significant challenge in the choice of treatment for patients with MBC who have failed one or more chemotherapy regimens. The repeated use of conventional anthracyclines is still believed to be limited by cumulative cardiac toxicity (Jones *et al*, 2006; Ryberg *et al*, 2008). Doxorubicin cardiotoxicity is dose dependent. The average incidence of doxorubicin-related cardiotoxicity is 5.1% in women who have received cumulative doses of 400 mg m^−2^ (Von Hoff *et al*, 1979; Swain *et al*, 2003). The incidence of cardiotoxicity exponentially increases with a cumulative dose of 500 mg m^−2^. This treatment-related cardiotoxicity, specifically linked to anthracycline use in these patients, has remained a challenge for physicians, and thus research in this area is increasing.

Because the majority of pretreated patients with MBC have been exposed to anthracyclines, either in the adjuvant or metastatic settings, there is a need for a cardiac tolerable and effective approach. Pegylated liposomal anthracycline formulations, such as pegylated liposomal doxorubicin (PLD, Caelyx; Schering-Plough, Kenilworth, NJ, USA), represent an attractive option in this setting. Single-agent PLD has repeatedly demonstrated comparable efficacy to doxorubicin, with less cumulative cardiac toxicity and less myelosuppression (Gabizon and Martin, 1997; O'Shaughnessy, 2003; Keller *et al*, 2004; O'Brien *et al*, 2004; Theodoulou and Hudis, 2004). Moreover, data suggest efficacy of PLD in the anthracycline rechallenge setting (Keller *et al*, 2004; O'Brien *et al*, 2004; Al-Batran *et al*, 2006a, 2006b; Trudeau *et al*, 2009).

There is no consensus about which parameters should be used in the decision regarding anthracycline rechallenge. Criteria clinicians need to consider include prior anthracycline treatment, prior taxane exposure, previous radiation, patient performance status, cardiac history and cardiac function, age and other co-variants. Two important factors, time since prior anthracycline therapy and cumulative dose of anthracycline, have been identified as predictive factors for the efficacy of anthracycline rechallenge in some studies; however, they have not been validated (Singal and Iliskovic, 1998).

Thus, we conducted a pooled analysis on individual patient data of MBC populations who received single-agent PLD rechallenge after previous exposure to conventional anthracyclines. The primary objective was clinical benefit rate (CBR). The secondary objective was to determine clinical factors that may predict the efficacy of PLD in anthracycline-pretreated patients with MBC.

## Materials and Methods

### Literature search and identification of studies

The aim of this study was to identify all relevant published prospective randomised clinical trials evaluating PLD as monotherapy in patients with MBC. A literature search was performed using databases (PubMed, CANCERLIT, the Cochrane Library and clinicaltrials.gov).

### Study selection

Eligible trials were prospective, in which patients received single-agent PLD for metastatic disease and included at least a subgroup of patients who had been pretreated with conventional anthracyclines. Four trials were identified from these searches for inclusion (Table 1; Keller *et al*, 2004; O'Brien *et al*, 2004; Al-Batran *et al*, 2006a, 2006b). The databases of these studies were provided by Merck, formerly Schering Plough Corp. The analysis was performed with the permission of the ethics committee responsible for our institution.

### Study objectives and data extraction

The primary end point, CBR, was defined as objective response, which included complete response, partial response or stable disease lasting longer than 6 months. Rechallenge with PLD was considered efficacious if the CBR exceeded 30%, whereas below 20% was considered inactive. The rate of 30% was considered clinically relevant taking into account the heavily pretreated population. *Post hoc* calculations provided 98% power to detect a CBR rate >30% (A'Hern, 2001).

Pre-specified clinical parameters, including baseline Eastern Cooperative Group (ECOG) performance status, number of previous chemotherapies, previous taxane, age, setting of prior anthracycline, cumulative dose of prior anthracycline, anthracycline-free interval and anthracycline resistance were evaluated for their association with the CBR, response rate (RR), progression-free survival (PFS) and overall survival (OS). Anthracycline resistance was defined as having disease progression while on anthracycline-based therapy for metastatic disease, or within 6 months of receiving adjuvant anthracycline treatment.

### Statistical analysis

Heterogeneity between studies was tested using the log-rank test for PFS and OS and the Fisher's exact test for CBR, RR and baseline parameters. The same statistical tests and models were applied to assess the univariate relationship between predefined variables and the primary and secondary end points. Furthermore, Cox proportional hazard models and logistic regression models were used for the multivariate analysis. In a secondary analysis, the univariate results were adjusted for baseline performance status, prior taxane therapy and number of previous chemotherapy regimens (these factors were found to be heterogeneous among the populations). Time-to-event curves was calculated by the Kaplan–Meier method. All *P*-values were two sided, with *P*-values <0.05 considered to indicate statistical significance.

## Results

### Characteristics of the patients

Of the 935 patients included in this analysis, 274 (29.3%) received PLD and had at least one prior conventional anthracycline therapy. The distribution of the predefined clinical parameters in the pooled population is shown in Table 2. The median age of the patients was 56 years (range, 29–87 years). Patients had a median of 3.5 prior treatment lines (range, 1–9), and 93.4% had at least two previous therapies (including chemotherapy and hormonal therapy). Prior anthracycline therapy was mostly administered in the metastatic setting, with prior anthracycline being adjuvant (14%), metastatic (46%) or in both settings (40%). Patients received a median of three cycles of PLD (range, 1–18), with a median dose of 83.8 mg per cycle and a mean cumulative dose of 311 mg (range, 25.5–1394 mg). There was significant heterogeneity between the studies regarding baseline ECOG performance status, taxane pretreatment and number of prior chemotherapy regimens. This heterogeneity was found to be attributed to the study by O'Brien *et al* (2004), which enrolled patients previously untreated in the metastatic setting (Table 1).

### Overall clinical benefit rate

Clinical benefit rate is shown in Table 3 (O'Brien *et al*, 2004; Keller *et al*, 2004; Al-Batran *et al*, 2006a, 2006b). The overall CBR was 37.2% (95% CI, 32.4–42.0). The lower boundary of the 95% confidence interval of the CBR observed was above the pre-defined 30% rate. The logistic regression model showed a higher CBR in the study by O'Brien (*P*<0.001) as compared with the study by Keller. In the pooled population, median PFS and OS were 3 (95% CI, 2.8–3.7 months) and 11.1 months (95% CI, 8.9–13.1 months), respectively.

### Outcomes according to clinical parameters: univariate analyses

Clinical benefit rate, RR, PFS and OS according to clinical parameters are shown in Table 4. There was no difference in CBR between patients who were considered anthracycline resistant and those who were not (40.5 *vs* 34.1% *P*=0.300). There also was no difference in CBR between patients who received prior anthracycline in the adjuvant setting (34.2%), in the metastatic setting (42.1%), or both settings (32.7%), *P*=0.332. There were no significant differences in CBR between patients who had low, medium or high cumulative doses of prior anthracycline at baseline (33.8 *vs* 37.0 *vs* 35.2%, respectively; *P*=0.913). A trend towards higher CBR was detected in patients who received PLD >12 months *vs* ⩽12 months since the end of their prior anthracycline therapy (40.7 *vs* 29.2% *P*=0.078). The adjustment for ECOG performance status, taxane pretreatment and number of prior chemotherapies revealed similar results (data not shown).

**Figure 1:**
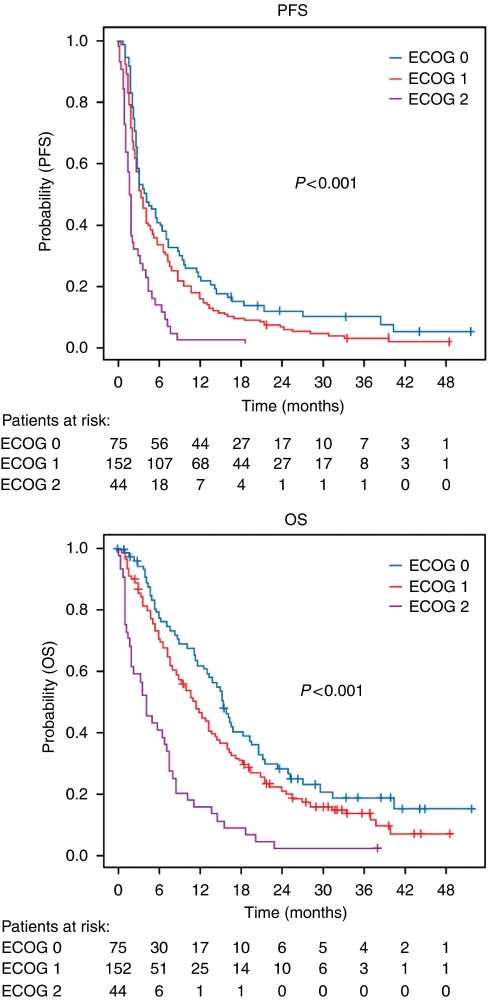
Progression-free and overall survival under pegylated liposomal doxorubicin therapy in patients previously treated with conventional anthracyclines Eastern Cooperative Oncology Group performance status.

Among clinical parameters not associated with prior anthracycline therapy, ECOG performance status was the strongest predictor of clinical benefit; CBR was 53.3, 35.5 and 18.2% in patients with ECOG performance status of 0, 1 and 2, respectively (*P*<0.001). In addition, a statistically significant higher RR, longer PFS and OS were observed for patients with ECOG performance status of 0 and 1 *vs* 2 (Table 4; Figure 1). Significantly higher CBRs were also observed in taxane-naive patients (53.0%) *vs* patients who received a previous taxane (30.1%), *P*=0.001, and in patients who failed only one therapy *vs* more than one therapy (1 *vs* 2 *vs* 3: 55.6 *vs* 24.6 *vs* 39.9%, respectively; *P*=0.024). Age was not a predictor of CBR. The univariate results regarding CBR were adjusted for ECOG performance status, taxane pretreatment and number of prior chemotherapies using a logistic regression model. The results were similar to the unadjusted models (data not shown).

### Multivariate analyses

All clinical parameters were included in a logistic regression model to determine their predictive effect on CBR. ECOG performance status was a strong predictor of CBR (*P*=0.038). The number of prior chemotherapies was no longer a significant predictor of CBR (*P*=0.192), and taxane pretreatment showed a non-significant trend (*P*=0.072). None of the other clinical parameters was statistically significant. In a multivariate Cox regression analysis, ECOG performance status also was a significant predictor of PFS (*P*=0.002) and OS (*P*<0.001). The number of prior chemotherapies was a significant predictor of OS (*P*=0.041).

## Discussion

This pooled analysis on the largest data set of MBC patients who were pretreated with an anthracycline evaluates the efficacy of anthracycline rechallenge using PLD. All patients had received conventional anthracyclines, and 72% had received prior taxane therapy. The majority were pretreated with more than one line of chemotherapy for metastatic disease. Therefore, most patients in this analysis were in an advanced and palliative course of their disease when they received PLD as an anthracycline rechallenge. Accordingly, we chose CBR as the primary end point for this patient population rather than RR or PFS (Ohorodnyk *et al*, 2009).

A clinical benefit is achieved when either an objective response or a long-lasting stable disease is documented. This reflects a direct and real effect of the rechallenge. Overall survival was not selected as the primary objective because it is influenced by other parameters such as further therapies and prognostic factors (Saad *et al*, 2010). The primary assumption of this study, that CBR >30%, was met in this analysis, where 37.2% of patients exhibited a clinical benefit from rechallenge with PLD (95% CI, 32.4–42.0). In the univariate analysis, patients with less exposure to prior treatment (i.e., one prior regimen or taxane naive) exhibited a statistically significantly higher CBR (53–56%) than patients that had received two or more prior regimens or prior taxane (25–36%). Moreover, patients with a favourable ECOG performance status (0 or 1) exhibited a statistically significantly higher CBR than patients with impaired ECOG performance status (2) (36–53 *vs* 18.2%, respectively).

In the multivariate analysis, ECOG performance status was determined to be the only independent predictor of the efficacy of anthracycline rechallenge with PLD. The ECOG performance status is widely used to quantify the functional status of cancer patients and is a common and consistent prognostic factor (Yates *et al*, 1980). However, the ECOG performance status can also predict the efficacy of a particular treatment, as patients with a better performance status are more likely to be compliant and maintain treatment duration and dose intensity (Nash *et al*, 1980; Sjöström and Blomqvist, 1996; Robain *et al*, 2000).

In contrast, no statistically significant differences in CBR were found in this pooled analysis for the following predefined parameters related to prior anthracycline therapy: (1) treatment setting for prior anthracycline administration, (2) anthracycline cumulative dose, (3) resistance to anthracycline and (4) anthracycline-free interval. These results translate into an expected significant efficacy from PLD rechallenge if the patient has an ECOG performance status of 0 or 1 (or conversely a poor efficacy if the patient has an ECOG performance status of 2), regardless of the number and type of prior therapies as well as prior anthracycline exposure. Notably, the lack of association between efficacy of anthracycline rechallenge and the parameters related to prior anthracycline therapy apply to the threshold definitions used in our study. It is possible that if alternative thresholds were used for the definitions of our parameters, then we would have encountered a different result in this analysis. Overall, the results of our study support the need to identify molecular-based predictive factors for anthracycline efficacy (Pritchard *et al*, 2008; Gianni *et al*, 2009).

In the recent literature, the rates of clinical benefit achieved with combination therapies in MBC patients varied between 39 and 80% in first-line patients, and 34 and 71% in patients previously treated for metastatic disease (Blum *et al*, 2006; Moulder *et al*, 2008; Silva *et al*, 2008; Osako *et al*, 2009; Ciruelos *et al*, 2010; Tan *et al*, 2010). Therefore, given the characteristics of our population and that single-agent therapy was used, the overall CBR of 37% observed in our study, is clinically meaningful and supports efficacy of an anthracycline rechallenge with PLD. This finding is in agreement with published reports that show activity and support the use of anthracyclines in anthracycline-pretreated patients (Gennari *et al*, 2004; Beslija *et al*, 2009; Katsumata *et al*, 2009; Krell *et al*, 2009). Notably, this analysis was not specifically designed to address the question of the first-line use of PLD after adjuvant anthracycline-based therapy. In the first-line setting, higher objective response and CBRs are observed after anthracycline rechallenge with PLD-containing combination chemotherapy (Overmoyer *et al*, 2005; Sparano *et al*, 2009; Trudeau *et al*, 2009).

The important question from findings of our pooled analysis is how these results fit into the evolving individualised field of chemotherapy for women with MBC. The ever-growing options for treatment coupled with biologically tailored research pose a challenge for clinicians. As a result, oncologists are increasingly shifting towards a more individualised treatment strategy based on factors such as patient and tumour characteristics, patient input and prior therapies. Our study justifies considering anthracycline rechallenge with PLD as one option for patients with MBC, who failed conventional anthracyclines or more agents in the adjuvant, metastatic or both settings, if the performance status of the patients is still favourable (i.e., ECOG performance status 0 or 1).

## Figures and Tables

**Table 1:** Trial characteristics

	**Al-Batran *et al* (2006a)**	**Al-Batran *et al* (2006b)**	**Keller *et al* (2004)**	**O'Brien *et al* (2004)**
Date first patient enroled	May 2000	October 2001	June 1997	June 1998
No. of patients				
Total/received PLD/received PLD after prior CAC	79/79/79	46/46/33	301/150/124	509/254/38
Study design	Non-randomised phase II	Non-randomised phase II	Randomised phase III with PLD *vs* comparator^a^	Randomised phase III with PLD *vs* doxorubicin
Study population	Women with at least 1 prior chemotherapy for MBC	Women with at least 1 prior chemotherapy for MBC	Women with taxane refractory MBC and ⩽2 prior chemotherapies for metastatic disease	Women, previously untreated for metastatic disease
Response assessment scale	WHO	WHO	NK	WHO
PLD schedule	50 mg m^−2^ every 4 weeks	40 mg m^−2^ every 4 weeks	50 mg m^−2^ every 4 weeks	50 mg m^−2^ every 4 weeks

Abbreviations: PLD=pegylated liposomal doxorubicin; MBC=metastatic breast cancer; CAC=conventional anthracycline; WHO World Health Organization; NK=not known.

a Vinorelbine or mitomycin C plus vinblastine.

**Table 2:** Distribution of predefined clinical parameters

	***N* (%)**
*Age*	
18–65 years	222 (81.0)
>65 years	52 (19.0)
	
*ECOG performance status*	
0	75 (27.4)
1	154 (56.2)
2	44 (16.0)
Unknown	1 (0.4)
	
*No. of prior therapies* a	
1	18 (6.6)
2	62 (22.6)
>2	194 (70.8)
	
*Previous taxane*	
Yes	198 (72.3)
No	66 (24.1)
Unknown	10 (3.6)
	
*Anthracycline-free interval*	
0–12 months	96 (35.1)
>12 months	150 (54.7)
Unknown	28 (10.2)
	
*Setting of prior anthracycline exposure*	
Adjuvant only	38 (13.9)
Metastatic only	126 (46.0)
Both	110 (40.1)
	
*Cumulative dose of prior anthracycline*	
<180 mg m^−2^	79 (28.8)
180–250 mg m^−2^	73 (26.7)
>250 mg m^−2^	88 (32.1)
Unknown	34 (12.4)
	
*Anthracycline resistance*	
Yes	118 (43.1)
No	138 (50.4)
Unknown	18 (6.5)

Abbreviation: ECOG=Eastern Cooperative Oncology Group.

a Including chemotherapy and hormonal therapy in the adjuvant and metastatic setting.

**Table 3:** Clinical benefit rate

	**Overall**	**Al-Batran *et al* (2006a)**	**Al-Batran *et al* (2006b)**	**Keller *et al* (2004)**	**O'Brien *et al* (2004)**
No. of patients	274	79	33	124	38
CBR, *n* (%)	102 (37.2)	30 (38.0)	8 (24.2)	37 (29.8)	27 (71.1)
95% CI	32.4–42.0	29.0–47.0	12.0–36.5	23.1–36.6	59.0–83.2
No CB, *n* (%)	170 (62)	49 (62)	25 (75.8)	85 (68.5)	11 (28.9)
NE, *n* (%)	2 (0.7)	—	—	2 (1.6)	—

Abbreviations: CBR=clinical benefit rate; CB=clinical benefit; NE=not evaluable for clinical benefit.

**Table 4:** Univariate analysis of outcomes according to clinical parameters

**Variable**	**CBR, *n* (%)** a	***P*-value**	**RR, *n* (%)** a	***P*-value**	**Median PFS (months)**	***P*-value**	**Median OS (months)**	***P*-value**
*Age*								
18–65 years	83/220 (37.7)		26/172 (15.1)		3.2		10.7	
>65 years	19/52 (36.5)	1	8/38 (21.1)	0.638	2.8	0.439	11.9	0.632
								
*ECOG performance status*								
0	40/75 (53.3)		17/59 (28.8)		4.1		15.5	
1	54/152 (35.5)		14/122 (11.5)		3.4		11.4	
2	8/44 (18.2)	<0.001	3/28 (10.7)	0.001	1.7	<0.001	4.1	0.001
								
*No. previous chemotherapies* b								
1	10/18 (55.6)		4/16 (25.0)		4.55		18.3	
2	15/61 (24.6)		8/55 (14.5)		2.9		9.3	
>2	77/193 (39.9)	0.024	22/139 (15.8)	0.415	3.1	0.405	10.9	0.041
								
*Previous taxane*								
Yes	59/196 (30.1)		17/146 (11.6)		2.8		9.9	
No	35/66 (53.0)	0.001	14/56 (25.0)	0.048	4.3	0.191	14.5	0.023
								
*Setting of prior anthracycline*								
Adjuvant	13/36 (36.1)		2/30 (6.7)		2.9		8.5	
Metastatic	53/126 (42.1)		24/102 (23.5)		3.7		11.9	
Both	36/110 (32.7)	0.332	8/78 (10.3)	0.050	2.6	0.949	11.2	0.439
								
*Cumulative dose prior anthracycline*								
<180 mg m^−2^	26/77 (33.8)		10/59 (16.9)		2.8		9.3	
180–250 mg m^−2^	27/73 (37.0)		8/55 (14.6)		2.8		11.9	
>250 mg m^−2^	31/88 (35.2)	0.913	8/73 (11.0)	0.697	3.1	0.616	12.3	0.333
								
*Anthracycline-free interval*								
0–12 months	28/96 (29.2)		9/74 (12.2)		2.6		10.7	
>12 months	61/150 (40.7)	0.078	20/120 (16.7)	0.429	3.2	0.253	11.9	0.892
								
*Anthracycline resistance*								
Yes	47/138 (40.5)		18/91 (19.8)		3.3		11.3	
No	47/116 (34.1)	0.300	14/104 (13.5)	0.237	3.0	0.604	11.1	0.611

Abbreviations: CBR=clinical benefit rate; RR=response rate (partial+complete response); PFS=progression-free survival time; OS=overall survival time.

a *n*=number of patients who were evaluable for predefined parameter and end point.

b Including chemotherapy and hormonal therapy in the adjuvant and metastatic setting.
